# Supplementary material for: Structure of the processive human Pol δ holoenzyme
Source: Nat Commun. 2020 Feb 28;11:1109. doi: 10.1038/s41467-020-14898-6 (PMC7048817; doi:10.1038/s41467-020-14898-6)
Supplement: Supplementary file 2 — Peer Review [file 41467_2020_14898_MOESM2_ESM.pdf]

Editorial Note: This manuscript has been previously reviewed at another journal that is not operating a transparent peer review scheme. This document only contains reviewer comments and rebuttal letters for versions considered at *Nature Communications.*

REVIEWERS' COMMENTS:

Reviewer #1 (Remarks to the Author):

After careful reading of all the answers to the points raised by the reviewers, I strongly believe that the authors have solved all the issues. The manuscript has improved substantially. Structures have been improved in resolution as well as in the clarity of the interpretations and validations.

This manuscript in its present form is a very important contribution to the field.

Reviewer #2 (Remarks to the Author):

The authors have satisfactorily addressed the issues raised in my initial review. This is important and interesting work, and I see no reason not to publish it in its current form

Reviewer #3 (Remarks to the Author):

This is an improved manuscript that addresses the points raised in my previous review. It can be published in its present form.
